# The invisible fish: hydrodynamic constraints for predator-prey interaction in fossil fish *Saurichthys* compared to recent actinopterygians

**DOI:** 10.1242/bio.014720

**Published:** 2015-11-24

**Authors:** Ilja Kogan, Steffen Pacholak, Martin Licht, Jörg W. Schneider, Christoph Brücker, Sebastian Brandt

**Affiliations:** 1 TU Bergakademie Freiberg, Geologisches Institut, Bereich Paläontologie/Stratigraphie, Bernhard-von-Cotta-Str. 2, Freiberg 09596, Germany; 2 Kazan Federal University, Institute of Geology and Petroleum Technologies, 4/5 Kremlyovskaya St., Kazan 420008, Russia; 3 TU Bergakademie Freiberg, Institut für Mechanik und Fluiddynamik, Lampadiusstr. 4, Freiberg 09596, Germany; 4 TU Bergakademie Freiberg, Institut für Numerische Mathematik und Optimierung, Akademiestr. 6, Freiberg 09569, Germany; 5 Sebastian Brandt arts, Im Schlufter 13, Kornhochheim 99192 , Germany

**Keywords:** *Saurichthys*, Ambush predators, Fossil fish, Locomotion, CFD, Palaeontology

## Abstract

Recent pike-like predatory fishes attack prey animals by a quick strike out of rest or slow movement. This fast-start behaviour includes a preparatory, a propulsive and a final phase, and the latter is crucial for the success of the attack. To prevent prey from escape, predators tend to minimise the duration of the interaction and the disturbance caused to surrounding water in order to not be detected by the prey's lateral line sensory system. We compared the hydrodynamic properties of the earliest fossil representative of the pike-like morphotype, the Triassic actinopterygian *Saurichthys*, with several recent pike-like predators by means of computational fluid dynamics (CFD). Rainbow trout has been used as a control example of a fish with a generalist body shape. Our results show that flow disturbance produced by *Saurichthys* was low and similar to that of the recent forms *Belone* and *Lepisosteus*, thus indicative of an effective ambush predator. Drag coefficients are low for all these fishes, but also for trout, which is a good swimmer over longer distances but generates considerable disturbance of flow. Second-highest flow disturbance values are calculated for *Esox*, which compensates the large disturbance with its extremely high acceleration performance (i.e. attacks at high speeds) and the derived teleostean protrusible mouth that allows prey catching from longer distances compared to the other fishes. We show CFD modelling to be a useful tool for palaeobiological reconstruction of fossil fishes, as it allows quantification of impacts of body morphology on a hypothesised lifestyle.

## Abstract (precis)

**Summary:** For the first time, the hydrodynamic properties of a fossil fish, *Saurichthys*, have been investigated using computational fluid dynamics. Low drag coefficient and small area of flow disturbance prove fast-start predatory adaptation.

## INTRODUCTION

Modern actinopterygian fishes exhibit a wide variety of body shapes, adapted for – and indicative of – a number of different lifestyles (e.g. Greenway, 1965). General appearance of the body, shape and placement of fins, structure and position of the mouth or type of dentition are only some of the characters that correlate with requirements of the habitat, locomotor performance, feeding habits and even times of activity during the day and night cycle. Such correlations are corroborated by numerous field observations, experimental and theoretical studies (e.g. Hobson, 1979; Keast and Webb, 1966; Lauder, 2006; Licht, 2009; Wainwright and Richard, 1995; Webb, 1984). Nonetheless, attribution of behavioural types to extinct animals based on comparison with extant forms must remain to some extent speculative, as long as no individual parameters of the fossils are taken into consideration (see Boucot and Poinar, 2010; Fletcher et al., 2014).


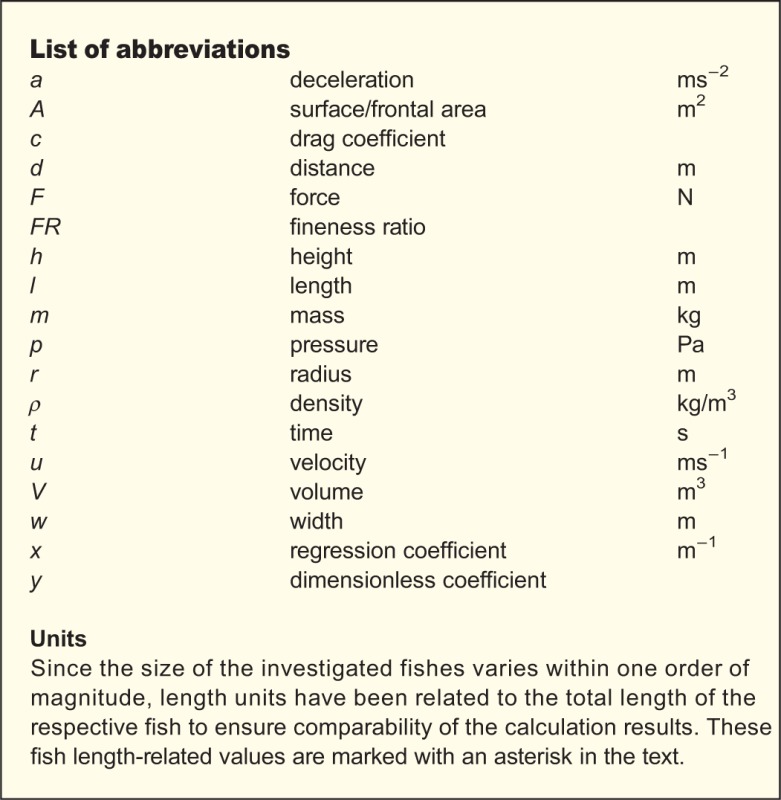


**Fig. 1.**
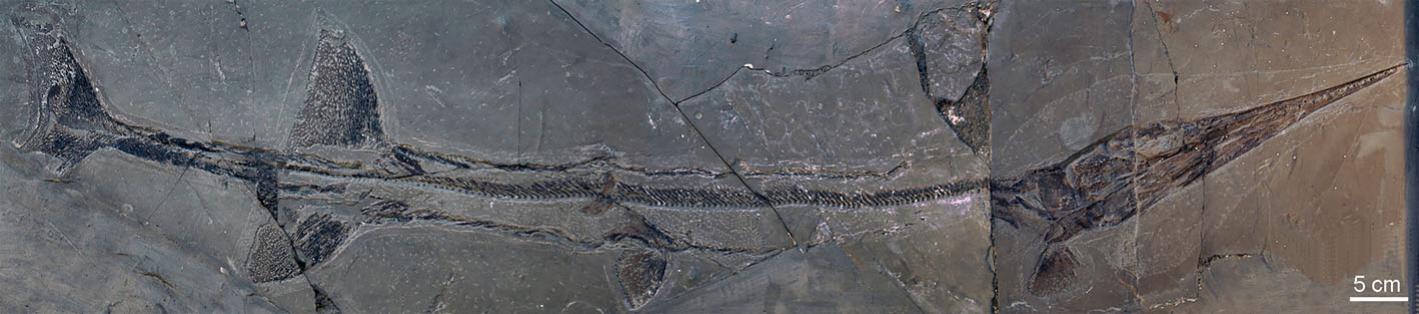
***Saurichthys deperditus* (****Costa, 1862****), a large Late Triassic saurichthyid from marine deposits of Austria.**

**Fig. 2.**
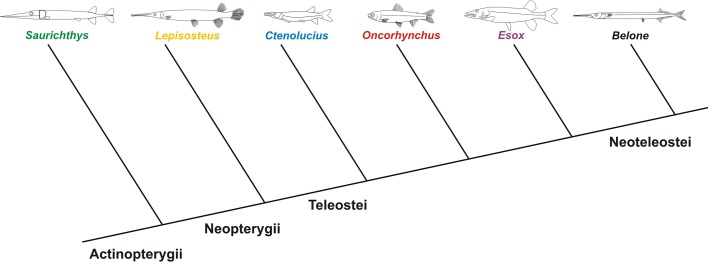
**Overview of the fish taxa incorporated in this study.** Phylogenetic relationships are summarised in the cladogram. The extinct *Saurichthys* is the basalmost genus included. Colours used here for the fish names are found in the following figures as well.

The term ‘pike-like predators’ is used to describe actinopterygians with elongated heads, long and slender bodies, posteriorly placed dorsal and anal fins and symmetrical tails. These mostly piscivorous fishes are not designed for long-term continuous swimming, but attack their prey by quick strikes out of rest or slow movement (also called fast-start or ambush predation). Fishes partly matching this body plan go back to the Palaeozoic, but the first typical representatives of such a morphotype appeared in the fossil record around the Permian-Triassic boundary and belonged to the ‘palaeopterygian’ family Saurichthyidae Owen, 1860 (*sensu* Stensiö, 1925). Soon after the End-Permian mass extinction, species of the genus *Saurichthys* Agassiz, 1834 (Fig. 1) radiated all over the globe, invading both marine and freshwater ecosystems. Saurichthyids ranged in length from few centimetres to more than 1.5 m, and at least the larger species are known to have been piscivorous (Kogan et al., 2014). Becoming rare in freshwater environments, saurichthyids retained the role of high-level consumers in the marine realm until their last representatives died out during the Jurassic (e.g. Romano et al., 2012). From that time on, however, similar morphologies independently arose in several actinopterygian clades (Fig. 2), raising the question whether or not they can be related to a similar lifestyle.

In fishes, fast-start predation (lunging) follows a well-known scheme that can be subdivided in distinct stages. Weihs (1973) identified a preparatory phase (I), during which the predator, when detecting the prey, changes from a stretched out position to an L or S shape; a propulsive phase (II), where the predator accelerates by quick movements in its posterior body half; and a final stage (III) of gliding or continued swimming (also called variable stage by some workers, e.g. Harper and Blake, 1991). Prey is caught in phase III (Frith and Blake, 1995; Webb and Skadsen, 1980).

The energetic costs of the fast-starting hunting tactics are high and need to be compensated with an increased success rate (e.g. Harper and Blake, 1988), which can be achieved, for instance, when the predator approaches the prey quicker than it can escape. Besides locomotor requirements to the fast-starting predator itself, perception of the approaching predator by the prey is crucial for the outcome of this interaction.

Fishes detect predators not only by vision, but also – and perhaps most importantly – by means of their lateral line sensory organ, which is sensitive to hydrodynamic signals (e.g. Bleckmann et al., 2004). To avoid being detected, an aquatic predator should therefore minimise flow disturbances caused by its movement. Experiments (e.g. Webb and Skadsen, 1980) demonstrate that fast-start predatory fishes reduce undulation of their body in the final phase of the strike and approach the prey largely without generating additional thrust.

As a first step towards the reconstruction of the possible hunting behaviour of the fossil actinopterygian *Saurichthys*, we examined the potential predator-prey interaction of this fish in comparison with several recent forms whose behaviour is known (Fig. 2, Table 1). For this purpose, we produced digital 3D surface models of a generalised *Saurichthys* and the recent pike-like actinopterygians *Esox lucius* (Linnaeus, 1758), *Belone belone* (Linnaeus, 1761), *Lepisosteus osseus* (Linnaeus, 1758) and *Ctenolucius hujeta* (Valenciennes in Cuvier and Valenciennes, 1849) and investigated their hydrodynamic properties in a digital (simulated) flow channel. Rainbow trout *Oncorhynchus mykiss* (Walbaum, 1792) was used as a control example for a predatory fish with a generalist shape.

**Table 1.**
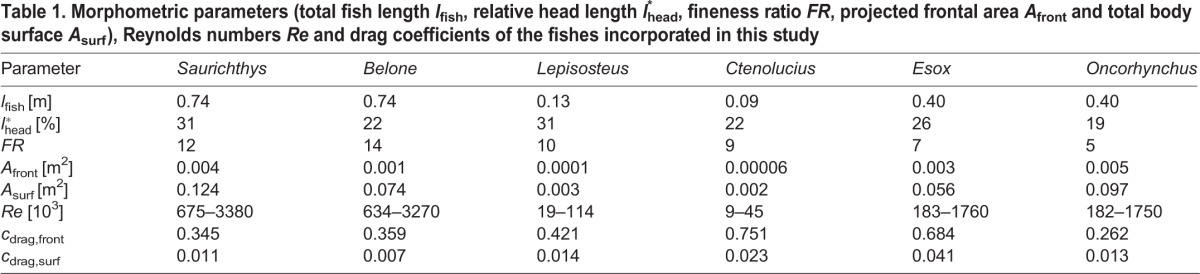
Morphometric parameters (total fish length *l*_fish_, relative head length *l*_head_^*^, fineness ratio *FR*, projected frontal area *A*_front_ and total body surface *A*_surf_), Reynolds numbers *Re* and drag coefficients of the fishes incorporated in this study

We used computational fluid dynamics (CFD) modelling to determine flow velocities around and pressure distribution at the surface of the fish body. Simulations have been performed with rigid fish models held stationary in a constant current (corresponding to a fish gliding at a constant velocity). This represents an approximation for phase III (*sensu* Weihs, 1973) of the fast-start attack where acceleration is terminated and the predator attempts to catch prey without producing additional thrust, so the actual speed may be nearly constant for a short time between the propulsive phase and the interaction with prey. To simulate predation in riverine and marine/lacustrine environments, different turbulence intensities have been implemented into the calculations.

## RESULTS

### Pressure

**Fig. 3.**
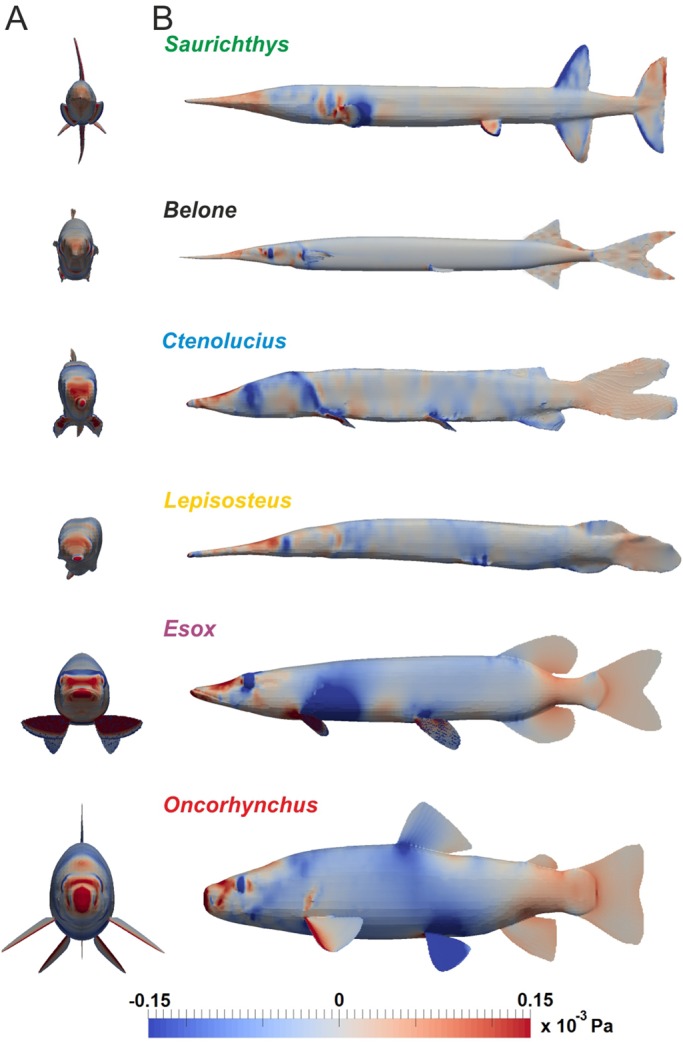
**Surface pressures.** Distribution of surface pressures (*p*=*p*_total_−*p*_dyn_) over the fish bodies at *u*_fish_=1 ms^−1^ in anterior view (A) and lateral view (B). Regions of low pressure are coloured in blue, high pressure areas in red.

The distribution of pressures over the fish body in a moving fluid is visualised in Fig. 3. Areas of elevated pressure are nearly absent in *Belone* and *Saurichthys*, small in *Lepisosteus* and *Ctenolucius* and large in *Esox* and *Oncorhynchus*. In general, the highest pressures are recorded in the anterior part of the head and at the leading edges of the paired fins, thus in the body portions facing the current. However, elevated pressures are also found at the caudal peduncle and over the caudal fin.

### Drag

All bodies moving through a fluid are confronted with resistance of the medium called drag. Total drag force *F*_drag_ is a combination of the pressure-induced drag *F*_pressure_ and the surface friction *F*_friction_.

**Fig. 4.**
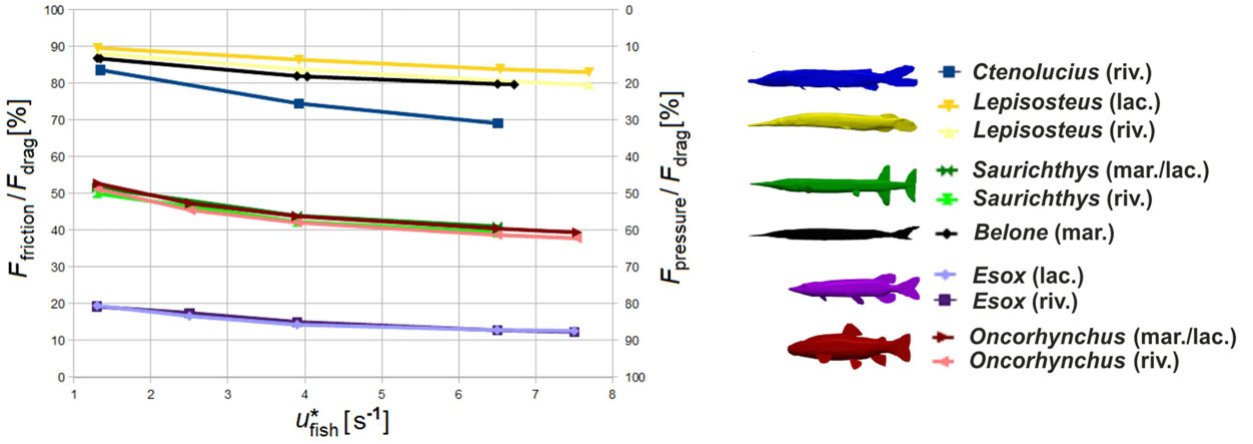
**Drag forces.** Speed-dependent variation of the components of the total drag force applied on the fish bodies, friction (left scale) and pressure-induced drag (right scale) over standardised fish velocity (u* in body length/second) in higher turbulent riverine (riv.) and less turbulent marine/lacustrine (mar./lac.) environments.

Whereas the total drag force *F*_drag_ increases with increasing speed and turbulence intensity (Table 2), the contribution of pressure-induced drag *F*_pressure_ and surface friction *F*_friction_ to total drag changes distinctly. Friction grows slower with higher velocities, so that drag is increasingly pressure-induced at higher swimming speeds (Table 2, Fig. 4). Among taxa, the percentage of friction varies from very low (*Esox*) to very high (*Lepisosteus* and *Belone*) (Fig. 4). The highest increase in pressure-induced drag compared to friction is noted for *Ctenolucius*.

**Table 2.**
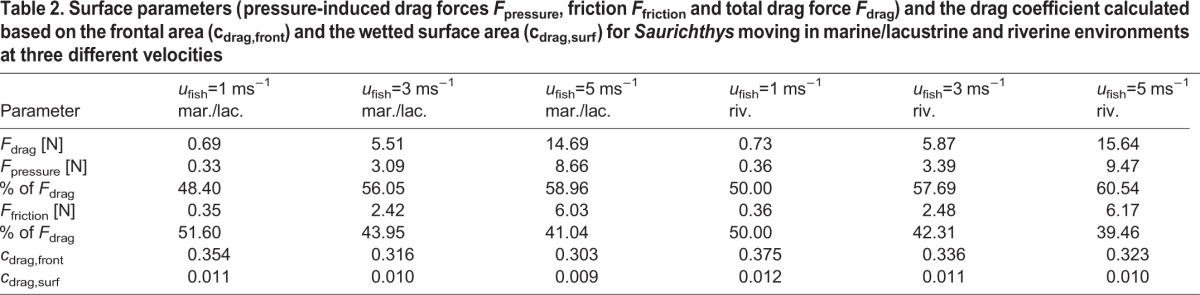
**Surface parameters (pressure-induced drag forces *F*_pressure_, friction *F*_friction_ and total drag force *F*_drag_) and the drag coefficient calculated based on the frontal area (c_drag,front_) and the wetted surface area (c_drag,surf_) for *Saurichthys* moving in marine/lacustrine and riverine environments at three different velocities**

A universally comparable drag measure is the drag coefficient *c*_drag_. According to different conventions, it can be calculated based either on the projected frontal area (*c*_drag,front_) or on the total body surface (*c*_drag,surf_). Both coefficients are nearly speed-independent and are lowest for *Belone*, *Saurichthys* and *Oncorhynchus* (Table 1). Drag coefficients are highest for *Esox* and *Ctenolucius*, and mostly higher in fluvial systems than in open waters (Table 2).

### Flow disturbance

**Fig. 5.**
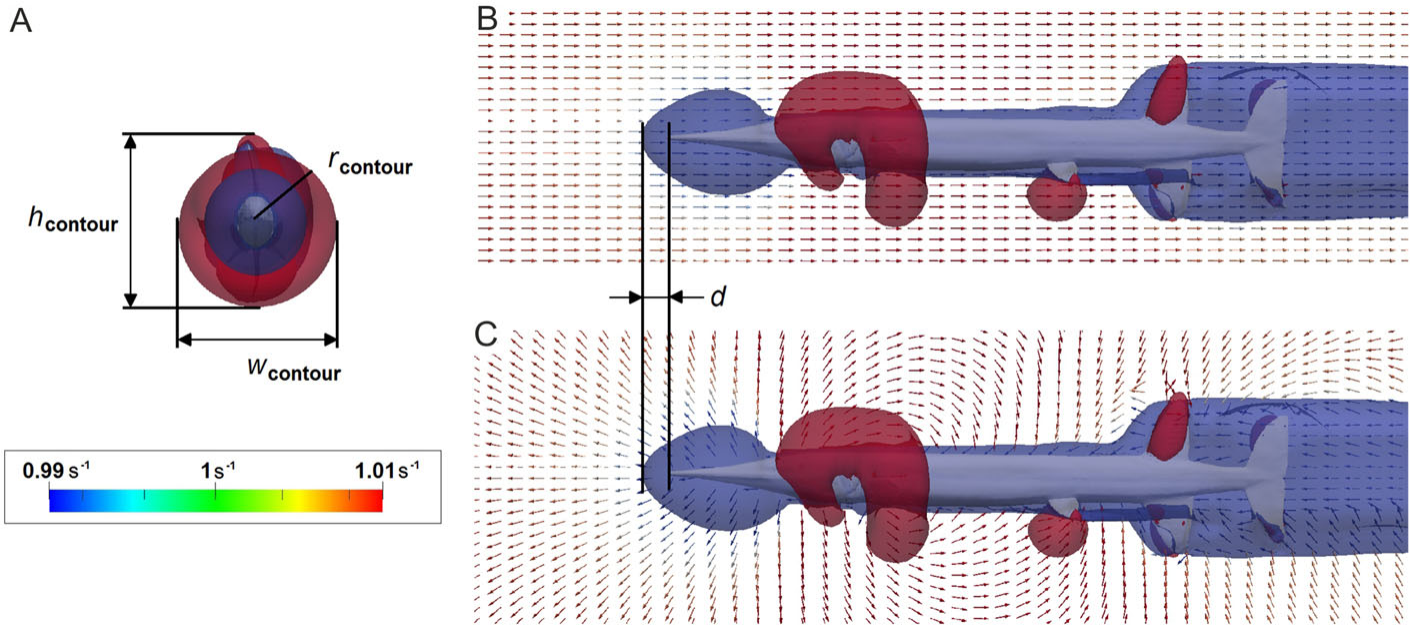
**Visualisation of the flow velocity disturbance around *Saurichthys* at *u*_fish_=1 ms^−1^.** (A) flow disturbance in anterior view; (B) disturbance of a constant current streaming around a static fish in lateral view; (C) disturbance caused by the fish moving through a static water body in lateral view. d, anterior expansion of the contour (snout distance); h, height; r, radius; w, width.

To analyse the hydrodynamic effects of an approaching predator, we quantified the disturbance of flow caused by the body of a fish moving through the fluid (Fig. 5). Flow disturbance can be expressed as variation of velocities in the flow channel where the fluid streams at a given inlet velocity *u*_fish_ (simulating the average swimming speed of the fish), while the fish body is held static (Fig. 5B), or as the circulation around the fish body when moving through a static fluid (Fig. 5C). Maximum disturbance is visualised in a contour plot generated by colouring areas of the fluid domain (shown in Fig. 5) where flow velocity *u*_local_ differs from the inlet velocity by at least 1%. Low velocity regions (*u*_local_≤99% · *u*_fish_) are coloured in blue and high velocity regions (*u*_local_≥101% · *u*_fish_) in red.

The dependency of flow disturbance on swimming speed and turbulence regime is shown in Table 3, using *Saurichthys* as an example. Disturbed area (expressed by the relative contour radius r*) is smaller at higher turbulence intensities, but virtually no effect is documented on the distance *d* from the tip of the snout to the anterior margin of the contour.

**Table 3.**
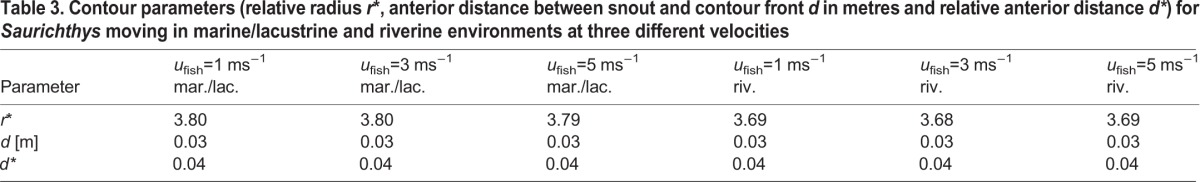
Contour parameters (relative radius r*, anterior distance between snout and contour front *d* in metres and relative anterior distance d*) for *Saurichthys* moving in marine/lacustrine and riverine environments at three different velocities

**Fig. 6.**
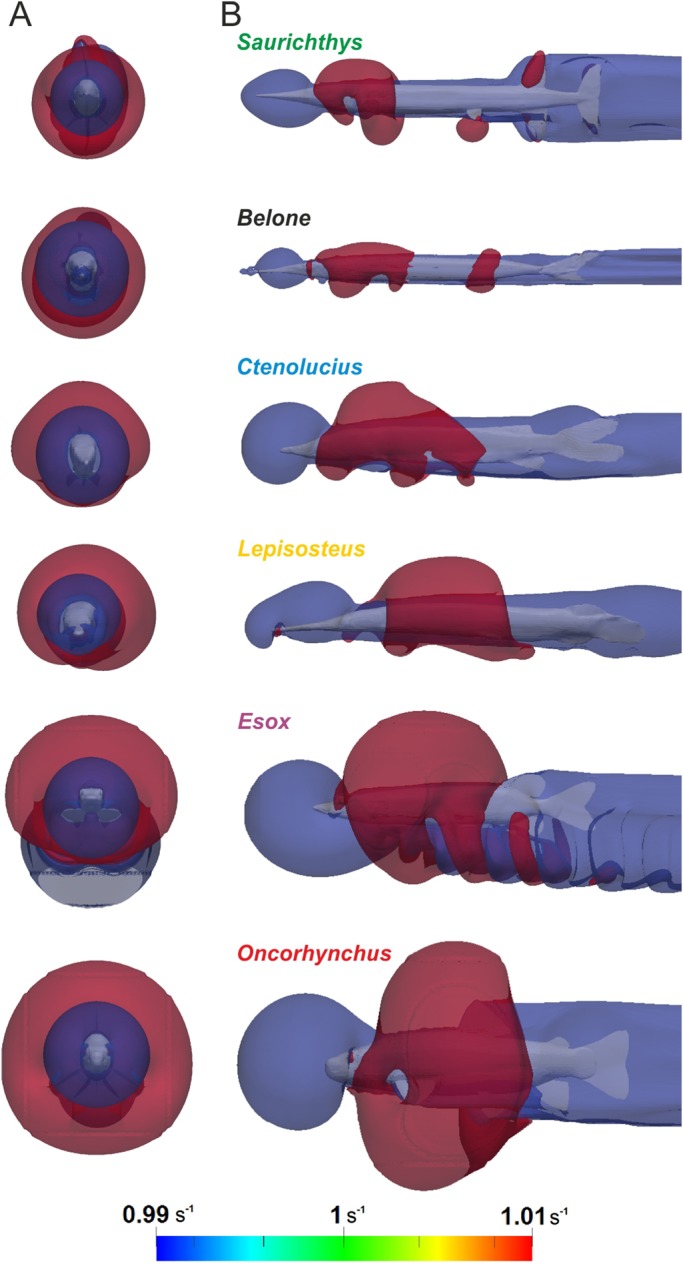
**Flow velocity disturbance around the fish bodies at *u*_fish_=1 ms^−1^.** Blue regions indicate low velocity areas (99% of swimming velocity) and red ones symbolize high velocity areas (101% of swimming velocity) in anterior view (A) and lateral view (B).

**Fig. 7.**
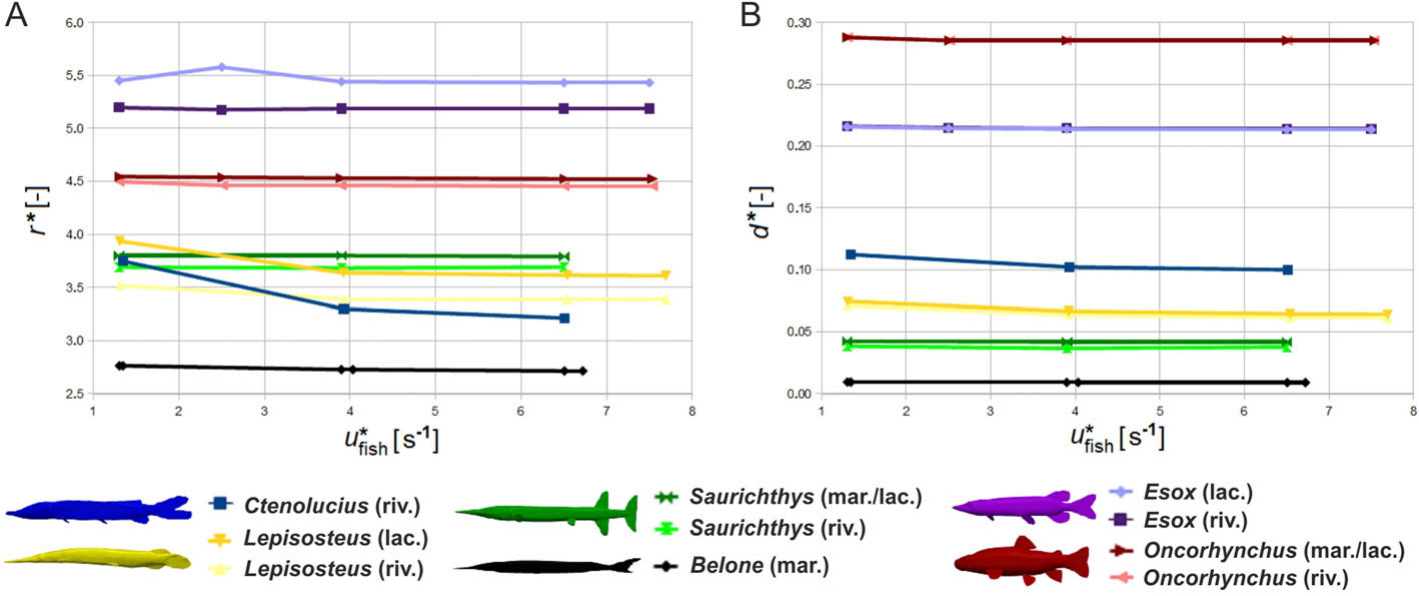
**Flow velocity disturbance.** Speed-dependent variation of the radius (A) and the anterior expansion (B) of the area of disturbed flow velocity around the fish bodies. u*, standardised fish velocity in body length/second; r*, contour radius/fish radius; d*, anterior contour expansion/fish length.

The largest flow disturbance area in the frontal plane is shown by *Esox*, followed by *Oncorhynchus*. In both taxa, the contour expansion comprises more than 4.5 times their own radius. *Belone* generates the minimal relative contour radius (Fig. 6A, Fig. 7A). The greatest differences in the contour radius between the less turbulent lacustrine and the higher turbulent fluvial environment were recorded for *Lepisosteus* and *Esox* (Fig. 7A). With increasing velocity, the relative area of flow disturbance slightly decreases for *Lepisosteus* and *Ctenolucius*, but remains nearly constant for the other fishes. Relative snout distance d* is lowest for *Belone* and highest for *Oncorhynchus*, followed by *Esox* (Fig. 6B), and is nearly unaffected by velocity (Fig. 7B). *Saurichthys* plots in the lower half of both diagrams close to *Belone* and *Lepisosteus*.

### Parametric studies

To evaluate the impact of environmental conditions on the hydrodynamic model, we performed additional calculations at different values of temperature, viscosity and density of the surrounding water. The temperature dependency of viscosity and density led to a triple parametric study that cannot be considered separately. In comparison with the initial calculations performed at 15°C, the results obtained for temperatures of 10°C and 20°C showed only minor deviation of less than 1%.

Additionally, a parametric study for turbulence intensity was made for several hunting domains (ideal-theoretic with nearly no turbulence, open marine/lacustrine, normal-fluvial and higher turbulent river). The results are compared in Table 4 and show only slight differences between ideal-theoretic and open marine/lacustrine habitat as well as between normal-fluvial and higher turbulent rivers at the considered parameters. Deviation remains constant irrespective of gliding velocity at which the calculations are performed.

**Table 4.**
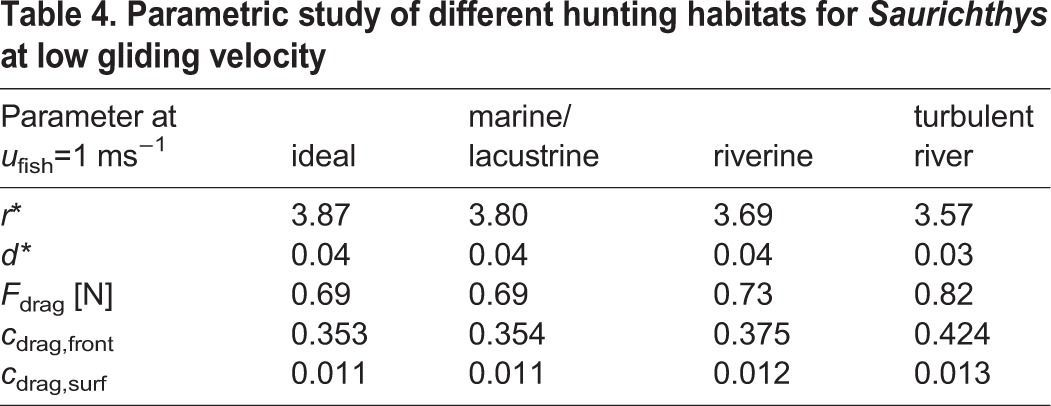
**Parametric study of different hunting habitats for *Saurichthys* at low gliding velocity**

### Morphometric predictability

**Fig. 8.**
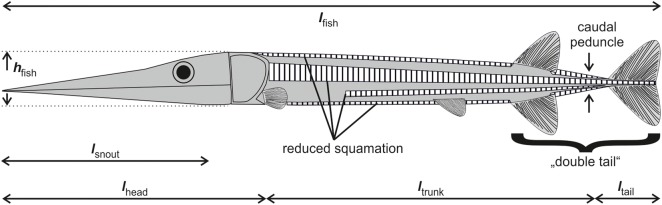
**General morphology of *Saurichthys* with some morphometric distances.** *h*_fish_, maximal height excluding fins; *l*_fish_, total body length; *l*_snout_, maximal length of the mouth gape; *l*_head_, head length; *l*_trunk_, trunk length from the hind margin of the operculum to the narrowest point of the caudal peduncle; *l*_tail_, length from the narrowest point of the caudal peduncle to the hind margin of the caudal fin. The dorsal, anal and caudal fins form the ‘double tail’ *sensu* Weihs, 1989.

We found no correlation between the various length distances measurable in the fishes (Fig. 8) and their hydrodynamic properties as summarised above. In contrast, the fineness ratio defined here as the ratio of total length to maximum height (excluding fins) is a good indicator for flow disturbance, pressure distribution and drag coefficient of the pike-like predators. Only the drag coefficient of trout is much lower than would be predicted from fineness ratio (Table 1).

## DISCUSSION

Body shape and lifestyle of animals are known to be correlated (e.g. Greenway, 1965). Although the investigated extant fishes with a pike-like body shape belong to different phylogenetic lineages (Fig. 2), they not only share morphological traits such as elongated skull, long and slender body, posteriorly positioned dorsal and anal fins, narrow caudal peduncle and symmetrical tail, but also exhibit considerable similarities in their hunting behaviour. They are considered fast-start predators, which attack mobile prey by quick, unexpected movements in order to prevent it from escape (Webb, 1984). Apart from the rapidity of the attack itself, this involves mechanisms for avoiding early discovery by the prey.

We assumed that successful fast-start predators should generate few flow disturbance to avoid being discovered by the prey's lateral line sensory system. Furthermore, as body undulations increase friction and disturbance of flow, predators reduce or cease undulation in the final phase of the strike and approach prey without additional thrust (e.g. Weihs, 1973; Webb and Skadsen, 1980). Consequently, one may expect that the drag coefficient of such predators is low, permitting them to reach the target easily once the propulsive phase is terminated. Indeed, disturbed area (normalized by fish length) is small for *Belone*, *Saurichthys*, *Lepisosteus* and *Ctenolucius* but large for both *Oncorhynchus* and *Esox*. The drag coefficients are also low for *Belone*, *Saurichthys* and *Lepisosteus*, but surprisingly high for *Esox* and *Ctenolucius.* In contrast, the generalist *Oncorhynchus* is characterised by a very low drag coefficient. According to our predictions, *Belone* and *Lepisosteus* should be the best-adapted recent forms for fast-start predation.

*Esox* is, nevertheless, seen as the iconic fast-starting predator in literature and even eponymous to the pike-like morphotype. When more flow disturbance is caused by its less streamlined body shape, pike should attack more quickly to prevent prey from escape. During acceleration, drag plays a minor role compared with fish mass, mass of water accelerated with the fish (added mass) and rate of acceleration (Webb, 1975), and so the higher drag coefficient of pike has less impact on fast-starting than muscle mass percentage (lower in forms with extensive body armour), lateral body profile or size and position of fins (Domenici and Blake, 1997; Weihs, 1989). Extensive experimental studies demonstrated the fast-start performance of pike to be superior to that of *Lepisosteus* (Webb et al., 1992) and trout (Harper and Blake, 1990), despite the pike's higher drag coefficient (see also Webb, 1988). Whereas in Harper and Blake's (1991) experiments, the prey – goldfish – attempted escape in most cases but were rarely successful, only 15% of fathead minnows used by Webb and Skadsen (1980) showed escape movements at all. This means that in spite of its relatively high flow disturbance, pike is quick enough to be successful in predation. The protrusible jaws of teleost fishes, primarily not developed in *Saurichthys* and *Lepisosteus* and secondarily lost in *Ctenolucius* and *Belone*, might be crucial for predatory ability in *Esox*.

Although a predator as well, the lifestyle of trout includes more continuous swimming, which is reflected in its generalist body shape. Trout causes high flow disturbance with the overall greatest anterior extension, but its streamlined body enables it to travel over long distances at low energetic costs (e.g. Tytell, 2007).

In terms of flow disturbance and drag, the fossil fish *Saurichthys* occupies a position intermediate to the extant neopterygians *Lepisosteus* and *Belone*, both of which nearly comply with our predictions for a successful fast-start predator. The model-derived hydrodynamic properties allow to evaluate the adaptation of *Saurichthys* for the fast-start hunting behaviour, which previously could only be suggested based on general morphology (Lombardo and Tintori, 2005; Rieppel, 1985; Tintori, 1990). We demonstrate that a hypothetical generalised saurichthyid caused moderate flow disturbance, especially low directly in front of the snout, and was able to maintain a relatively long final phase of the strike thanks to its low drag coefficient. Differences in squamation, vertebral column and fin morphology certainly had further impacts on the swimming behaviour of different *Saurichthys* species, which shall be quantified in forthcoming studies.

Environmental factors like water temperature, density and viscosity were found to have little effect on fish hydrodynamic properties when varied within a natural range. The only considerable deviation was caused by different turbulence intensities corresponding to open marine, lacustrine or riverine habitats. These especially influence drag forces as well as contour radius of the created flow disturbance. For instance, we noted a decrease of relative contour radius of *Saurichthys* with increasing turbulence but an increase of drag forces. So the prey may recognize the predator at a later moment but the predator needs a greater amount of energy to catch it in a turbulent environment. However, the general trends presented above remain constant throughout the turbulence regimes.

Our results show that numerical hydrodynamic modelling is a good tool to estimate the performance of fossil aquatic animals and to test hypotheses concerning their lifestyle. Furthermore, the procedure presented here is a low-cost method in comparison with experimental studies. Refinement of the calculation routines and inclusion of more morphological data as well as further taxa will lead to more detailed and quantifiable outcomes. Applied to fast-start predatory fishes, modelling of the acceleration phase and implementation of motion functions shall allow precise reconstructions of the complete strike movement, which is crucial to understand the evolution of predator-prey interactions.

## MATERIALS AND METHODS

### Considered taxa

#### Saurichthys

51 species are currently considered valid in the family Saurichthyidae, one of which is of Late Permian, 48 of Triassic and two of Early Jurassic age (see Romano et al., 2012, for an overview, and Maxwell et al., 2015; Tintori, 2013; Tintori et al., 2014; Werneburg et al., 2014 and Wu et al., 2015 for newest taxa). About one third of the Triassic species are documented by material sufficiently complete as to allow the reconstruction of the whole skeleton. For the purpose of this work, a generalised saurichthyid morphology was required, which is compiled from the best-known species of Early, Middle and Late Triassic. Besides several schematic drawings, whole-body restorations of Triassic saurichthyids have been published by Griffith (1977), Kogan et al. (2009) and Rieppel (1985).

Synapomorphic characteristics of the genus *Saurichthys* can be summarised as follows (Figs 1, 8): long and slender body (fineness ratio *sensu* Maxwell and Wilson, 2013, up to 20); elongate head usually accounting for 1/4 to 1/3 of the total body length; pelvic fins placed near the middle of the trunk; dorsal and anal fins symmetrical to each other and placed in the middle of the distance between the pelvics and the caudal fin; the latter externally and structurally symmetrical, having equal epaxial and hypaxial lobes separated by the vertebral column that proceeded straight to the posterior margin of the tail (abbreviate-diphycercal tail, Brough, 1936); narrow caudal peduncle, stiffened by interlocking scutes along the dorsal and ventral midlines; and a specialised, usually strongly reduced squamation (Gardiner, 1960; Griffith, 1959, 1962; Maxwell and Wilson, 2013; Rieppel, 1985, 1992; Romano et al., 2012; Stensiö, 1925; Woodward, 1888, 1890, 1895). The axial skeleton of *Saurichthys* lacks ossified vertebral centra and consists of a persistent notochord flanked by up to 200 pairs of neural arches dorsally and a usually lower number of haemal arches ventrally. In several species, the vertebral column is stiffened by long processes directed anteriorly and posteriorly to the neural arches (Tintori, 1990, 2013). Furthermore, many forms possessed unsegmented or rarely segmented fin rays, indicating increased stiffness of the fins (Romano et al., 2012; Schmid and Sánchez-Villagra, 2010).

#### Recent forms

*Lepisosteus osseus* (Ginglymodi: Lepisosteiformes) lives in rivers and lakes of North America and is one of the rare recent fishes whose body is covered by rhombic ganoid scales. Lepisosteids feed mainly on fish, but can also include crustaceans, higher vertebrates and carrion in their diet (Kammerer et al., 2006). They approach their prey rather slowly before attacking it by a quick lunge (Kammerer et al., 2006; Porter and Motta, 2004).

*Ctenolucius hujeta* (Ostariophysi: Characiformes) is found in rivers of equatorial South America, where it preys on small fish and occasionally decapods. *Ctenolucius* has bright silver coloured skin and hides in small inlets waiting for prey that it catches with a quick strike (Vari, 1995).

*Belone belone* is a European representative of the needlefishes (Teleostei: Beloniformes) and lives in surface waters of the North Sea, the Black Sea, the Azov Sea, the Mediterranean and its adjacent regions. Belonids are piscivorous and use a similar hunting strategy, performing relatively quick strikes over short distances (Collette, 2003; Porter and Motta, 2004).

*Esox lucius* (Teleostei: Esociformes) is the emblematic ambush predator whose prey capture behaviour has been extensively studied (e.g. Harper and Blake, 1991; Frith and Blake, 1995; Rand and Lauder, 1981; Schriefer and Hale, 2004). Species of *Esox* are found in fresh waters of the northern hemisphere and are essentially piscivorous (Bregazzi and Kennedy, 1980) and hide in vegetated areas of the water body waiting for prey that they attack by quick strikes.

*Oncorhynchus mykiss* (formerly called *Salmo gairdnerii*) is a widespread salmoniform teleost with a generalist morphology (Greenway, 1965), able to hold position in streams, to perform fast-start escapes, but also to migrate over long distances (Przybilla et al., 2010; Tytell, 2007; Webb, 1976). In nature, the diet of trout largely depends on age and available food, being composed in varying proportions of benthic macroinvertebrates, terrestrial insects, fish and even plants (Di Prinzio et al., 2013; Rikardsen and Sandring, 2006).

#### Morphometrics

For the *Saurichthys* model and the recent fishes investigated, we recorded several morphometric measurements, such as head length, total body length and maximum height (Fig. 8, Table 1). Head length is measured from snout tip to the posterior margin of the operculum. Additionally, we considered the fineness ratio (*FR*) defined as the relationship between total length and maximum height (without fins). We furthermore calculated fish volume and mass out of the 3D geometry (Table 6).

### Obtaining geometries

#### *Saurichthys* plastic model

**Fig. 9.**
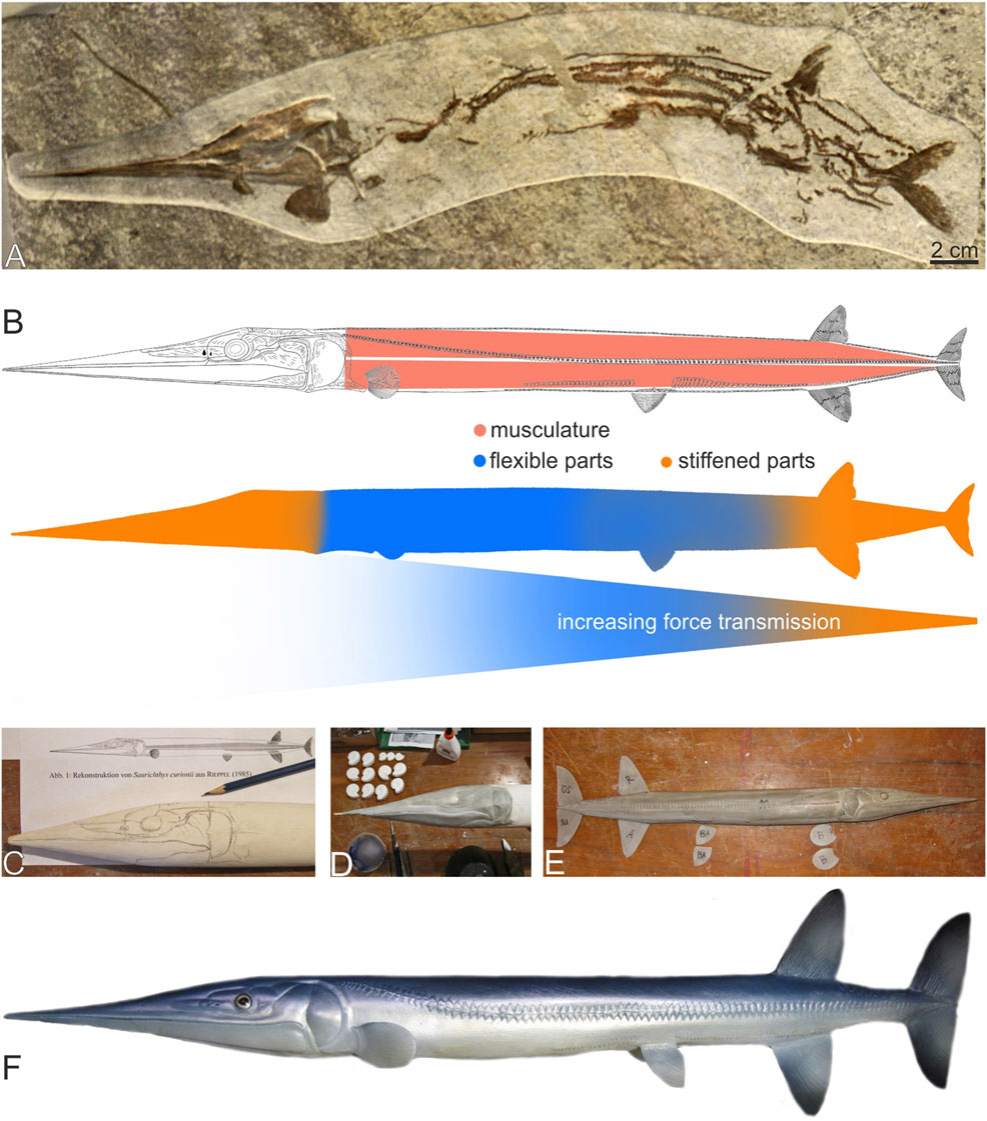
**Steps of plastic rebuilding of a generalised *Saurichthys*.** (A) a Middle Triassic *Saurichthys curionii* (Bellotti, 1857) from Monte San Giorgio, Switzerland; (B) presumed distribution of muscles, soft and stiffened regions, based on Rieppel's (1985) reconstruction of *S. curionii*; (C) sketch of the cranial elements on a model of *Saurichthys* head; (D) modelling of the snout and head structures; (E) application of paired fins; (F) completed *Saurichthys* plastic model.

The proportions mentioned above for *Saurichthys* are in good agreement with those of the recent garfish *Belone belone*, which therefore was used as a living control form. The lateral outline of *Saurichthys*, based on the published restorations and own observations (I.K., S.B.), was combined with shape data from three-dimensionally preserved saurichthyid skulls and supplemented with the garfish soft part morphology to obtain a true to scale *Saurichthys* trunk model executed in rigid foam (Fig. 9). Morphological details such as skull bones, muscles and scales have been formed in modelling resin and plastically applied in several steps. Special attention was paid to the configuration of the head, the squamation and the skin. Fins have been modelled separately, and their position was marked on the trunk model according to scientific reconstructions (Kogan et al., 2009; Rieppel, 1985). Negative silicone casts of all parts were prepared, allowing to reproduce the surfaces in epoxy resin. These could now be combined into a hollow body with an ultralight metallic core wire. Since the material used is thermally plastic, the obtained models can be deformed depending on the required position. For display purposes, models can be airbrushed in colours inferred from recent examples and covered with protective lacquer.

#### Digitalisation

The *Saurichthys* model was digitalised using a mobile MicroScribe 3D-Scanner from the Technical University of Dresden and the rendering software MicroScribe Utility for creating a point cloud of the scan data. Applying a Delaunay triangulation algorithm on this data field led to a stereo lithography (STL) surface consisting of a finite number of triangles (Pacholak et al., 2014). Specimens of *Belone*, *Lepisosteus* and *Ctenolucius* were scanned by CreaForm using a mobile HandyScan3D and were transformed the same way into STL data. The geometries of rainbow trout and northern pike were taken from the Online Toucan Virtual Museum of the Toucan Corporation Japan (http://www.toucan.co.jp/3DCG/3ds/FishModelsE.html) and converted from Tank3 Demo Files into STL with NURBS Modelling for Windows (Rhinoceros), covering gill openings and closing the mouth, as no surface holes or unnatural rough edges are allowed to fulfil the circulation condition for fluid dynamics.

Additionally, deformations of scanned specimens like back bone bending caused by conservation or adverse storing could be undone by applying a reverse motion algorithm onto the surface models in MATLAB (The Mathworks Inc.). This method unbends a deformed vertebral column back to prone position under consideration of the length consistence criteria so that both the curved and the straightened model have the same length after editing. Afterwards the unbent surface points are calculated using their relative position to the back bone.

### Numerical methods

#### Steady gliding

**Fig. 10.**
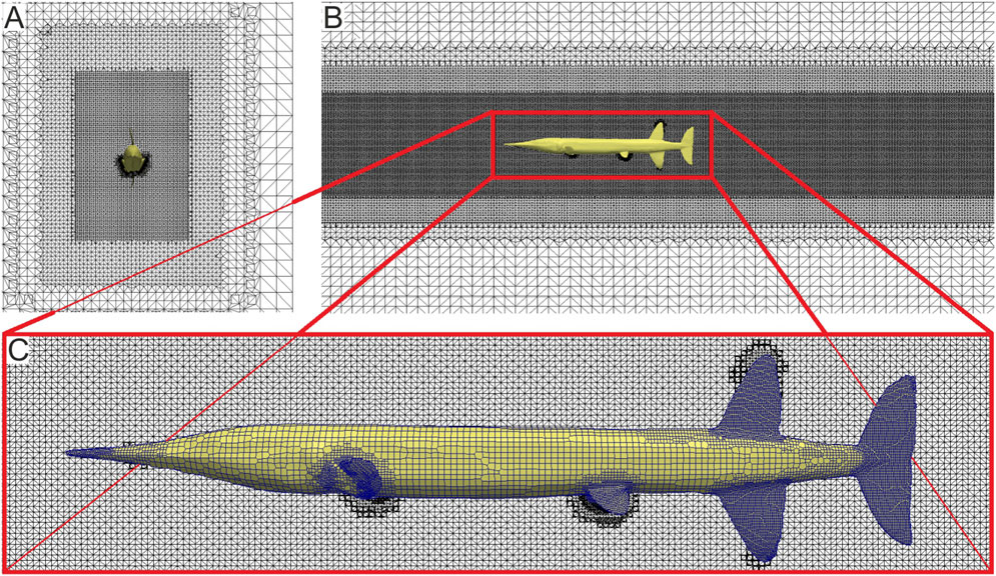
**CFD calculation mesh.** Discretised flow channel around digitalised *Saurichthys* model with a relative size of five fish long, nine fish high and seven fish wide in anterior view (A) and lateral view (B). Each grid cell contains a spatial solution of the NS equation, so the resolution of the grid has a finer core near the surface of *Saurichthys*. (C) Discretisation of the model surface to calculate surface forces.

The basic study of hydrodynamic properties of the fish bodies involves their interaction with the fluid at constant swimming speeds. We simulated gliding at constant speeds numerically by placing rigid surface models of the fishes into a digital flow channel. The latter approximates the fish's natural hunting environment that can be characterised by temperature, density and turbulence intensity of the surrounding water. Up to six different velocities were investigated for each fish species. When fluid parameters are held constant, the distribution of pressures and stream velocities over the fish body depends on body shape. To calculate the pressure-induced forces (lift and drag) and the viscous force (friction) out of pressure and stream velocity on the fish surface, the governing equations of fluid dynamics, the Navier−Stokes Equations (NS), mass conservation as well as momentum conservation:
(1)
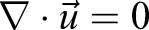

(2)
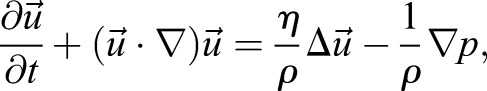


where *p* is pressure, 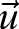 is fluid velocity, *ρ* and *η* are density and dynamic viscosity of water at time *t*, have to be solved numerically. Unfortunately, an analytic solution for NS with turbulence models (here we used the *k*-*ω*-SST model) describing finer vortex structures and velocity development still doesn't exist. So the circulation area surrounding the fish model had to be divided into a finite number of grid cells (shown in Fig. 10 for about 5 million cells totally) for a discrete spatial solution. Therefore an outer hexahedral mesh and a finer inner mesh adapted to the fish's shape was created. Surface layers were added along the fish surface to enhance the wall resolution. The spatial discretisation is also applied to the fish surface model to calculate surface pressures (Fig. 10C). In each cell, the NS is solved using numerical approximation functions for the pressure and velocity field. The according boundary conditions can be found in Table 5.

**Table 5.**
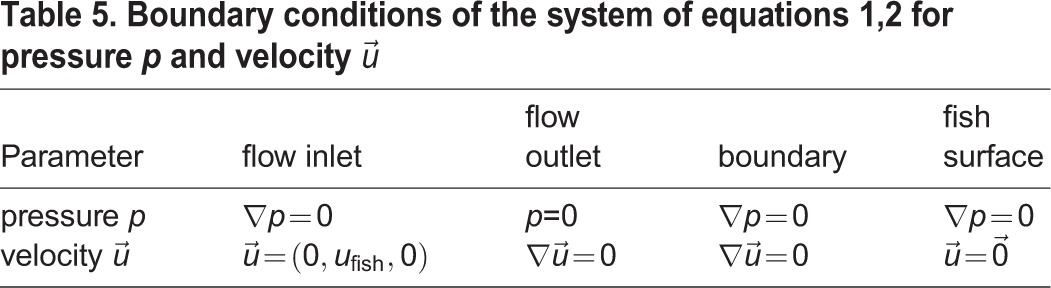
**Boundary conditions of the system of equations 1,2 for pressure *p* and velocity** 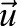

Calculations were performed by OpenFOAM using the finite volume method with second-order discretisation in space at a stationary case (e.g. Ferziger and Peric, 2002; Versteeg and Malalasekera, 2007) in species-specific flow channels spanning five times the length, nine times the height and about seven times the width of each fish, with a water temperature set at 15°C and standard turbulence intensity values for a (less turbulent) marine/lacustrine and a (higher turbulent) fluvial environment, according to the natural habitats of the studied fishes (see Sukhodolov et al., 1998).

#### Post-processing

While only the distribution of pressures and flow velocities is directly obtained from the calculation, several other parameters can be derived from these results. These include the magnitude and shape of flow disturbance around the fish, friction and pressure-induced drag force and the drag coefficients.

The area within which flow velocity of the water is disturbed by 1% or more compared with *u*_fish_ (inlet velocity of the flow channel that corresponds to the average fish velocity) is expressed by its envelope (contour). To estimate flow disturbance around the fish body, the radius of the greatest expansion of the contour (shown in Fig. 3) *r*_contour_=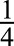
(*h*_contour_+*w*_contour_) is divided by the mean fish radius *r*_fish_=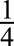
(*h*_fish_+*w*_fish_):
(3)
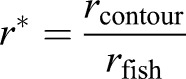
where *h* is height and *w* width. The distance *d* between the tip of the fish snout and the anteriormost point of the contour is of greatest interest because this is the distance at which a frontally attacked prey would detect the approaching predator.

Drag is the force applied to any type of body moving through a fluid. It can be expressed as
(4)
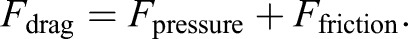
This drag includes pressure-induced drag forces *F*_pressure_ and surface friction *F*_friction_. *F*_pressure_ follows from the distribution of pressure values *p*_*i*_ over the fish body, which in turn are calculated incrementally for small, user-defined surface areas *A*_*i*_:
(5)
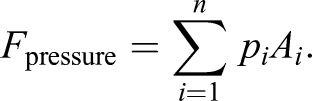
*F*_friction_ is approximated considering the surface roughness and thus the fluid adhesion. Surface roughness is derived from the scan data and expressed by the grid mesh on the body surface; the actual scan resolution does not allow for incorporation of finer surface structures in the model. Remaining forces from surface waves or interactions with other objects can be neglected as well as the lift forces. It can be assumed that fishes are able to compensate the lift through control of their swim bladder.

Out of equation 5 the drag coefficient
(6)
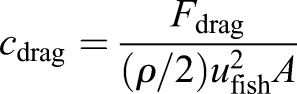
is calculated, where 
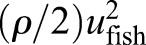
 is the dynamic pressure that is induced through the inlet velocity *u*_fish_ and fluid density *ρ*, and the specified area *A* is the projected frontal area (*A*_front_ for calculating *c*_drag,front_) or the total wetted surface (*A*_surf_ to determine *c*_drag,surf_), respectively.

#### Estimation of gliding ability

When predators cease undulating and approach their prey gliding in the final stage of the strike, it may be interesting to estimate how far they can glide before the movement is stopped by drag forces. Calculations of gliding distance have proven difficult in our simulation setting, but drag coefficient and gliding deceleration can be used as appropriate proxies.

Deceleration of a gliding movement without propulsion results from the division of drag forces *F*_drag_ by the fish mass *m*_fish_: *a*_drag_=(*F*_drag_/*m*_fish_). Taking into account that most fishes accomplish neutral buoyancy using their swim bladder, we assume that mean density of a fish with swim bladder must be equal to that of water and density of the fish excluding swim bladder should be significantly higher. Weighing the recent fish specimens at our hand led to establishing the following approximation for the fish mass:
(7)
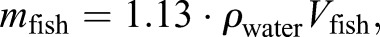
where *V*_fish_ is the volume of the fish and *ρ*_water_ the density of the replaced water. Fish volumes are determinable from the available surface models (Table 6).

**Table 6.**
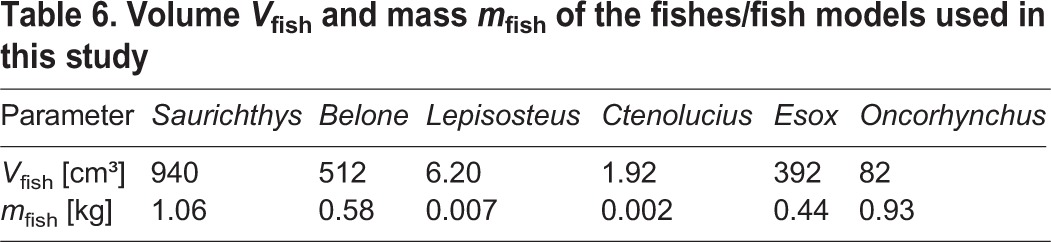
**Volume** *V*_fish_ **and mass** *m*_fish_ **of the fishes/fish models used in this study**

Deceleration is speed-dependent and can be derived from the model only for the initial velocities incorporated in the calculation. The relationship between velocity and deceleration *a*_drag_ shows an exponential behaviour and can be approximated by the regression:
(8)
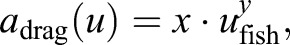


where *x* and *y* are variable components specific to each fish's body shape. Since *y*≈2 for all investigated species, *x* is given in m^−1^.

**Fig. 11.**
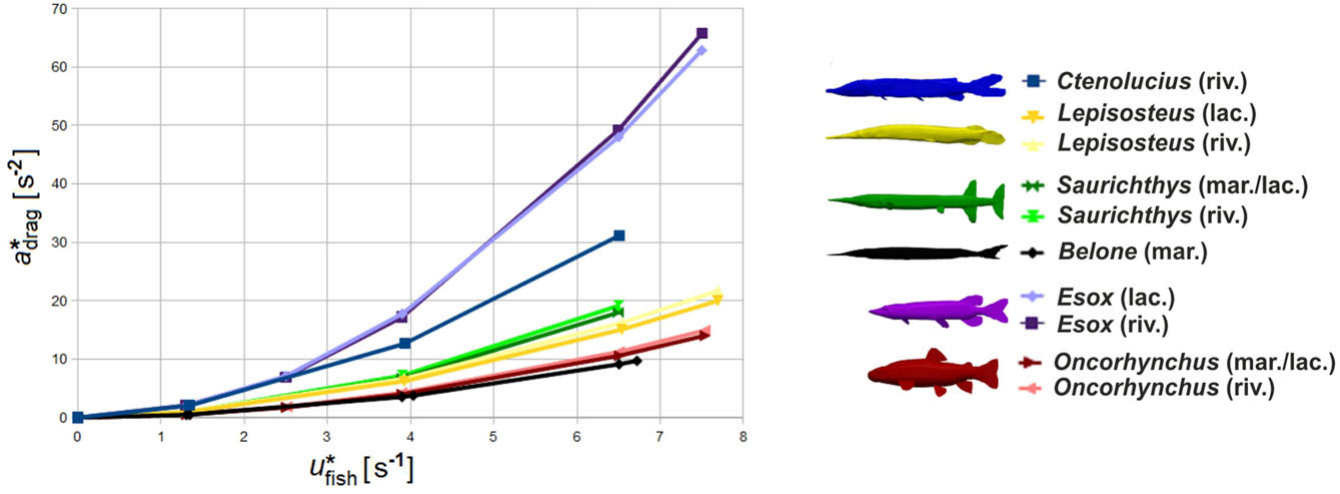
**Gliding abilities.** Variation of the gliding deceleration 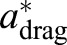 over standardised fish velocity (u* in body length/second) in higher turbulent riverine (riv.) and less turbulent marine/lacustrine (mar./lac.) environments.

With increasing swimming speed, *Esox*, *Ctenolucius* and *Saurichthys* exhibit the highest deceleration values (Fig. 11). Lowest gliding deceleration is noted for *Belone*. Deceleration is somewhat higher in the riverine environment. Calculation results for *Saurichthys* are given in Table 7.

**Table 7.**
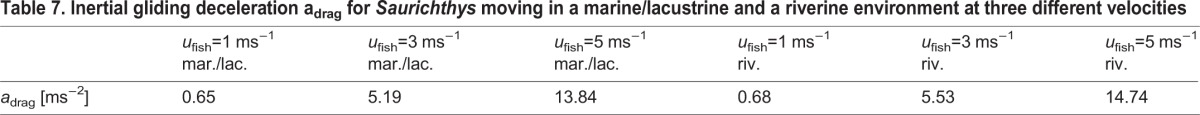
**Inertial gliding deceleration a_drag_ for *Saurichthys* moving in a marine/lacustrine and a riverine environment at three different velocities**
